# The challenges of managing malignant head and neck tumors in a tropical tertiary health center in Nigeria

**Published:** 2011-11-06

**Authors:** Adoga Adeyi, Silas Olugbenga

**Affiliations:** 1 Otorhinolaryngology Unit, Department of Surgery, Jos University Teaching Hospital, PMB 2076, Jos, Plateau State, Nigeria; 2 Department of Pathology, Jos University Teaching Hospital, PMB 2076, Jos, Plateau State, Nigeria

**Keywords:** Management, challenges, malignant tumors, head, neck, Nigeria

## Abstract

**Introduction:**

In developing countries, factors such as late patient presentation, inaccessible and limited health facilities contribute to the poor outcome in the management of patients with head and neck malignant tumors. This study presents the challenges faced by the otorhinolaryngologist in our environment in the management of patients with head and neck malignant tumors.

**Methods:**

This is a ten-year retrospective chart review of patients with histopathologically confirmed head and neck malignancies in the Jos University Teaching Hospital, Jos, Nigeria.

**Results:**

Eighty nine patients, with male predominance (gender ratio of 2.7:1) aged between 23 and 78 years had head and neck malignancies. Eighty eight (99%) patients had histopathological diagnosis. Most patients were from rural areas and had seen the herbalist prior to presentation. Thirty four (38.2%) patients were in the 4th decade of life. Eighteen (20.2%) patients presented within 6 months of onset of illness and 71 (79.8%) patients after 6 months with 38/89 patients having cervical lymphadenopathy at presentation. Four (4.5%) patients were able to afford CT scan. Twenty eight deaths were recorded. Ten patients were lost to follow-up.

**Conclusion:**

The challenges faced in managing patients with head and neck malignant tumors in our environment are enormous but surmountable. Therefore urgent efforts should be made by health workers and government to ensure a better outcome for these patients.

## Introduction

The management protocol for patients with head and neck malignant tumors involves a full history of the disease and clinical examination supplemented by investigations such as computerized tomographic (CT) scan and magnetic resonance imaging (MRI). Further assessment with fine needle aspiration biopsy (FNAB) and an examination under anesthesia helps to provide a histological diagnosis enabling the commencement of early and proper treatment [1–3].

Both tumor and patient factors affect the final outcome of patients with head and neck malignant tumors. These factors include age of patient, stage of tumor at presentation and the presence or absence of concomitant medical conditions.

The interplay of several factors in our environment contributes to the poor outcome in the management of patients with head and neck malignant tumors. These factors are late patient presentation, inaccessible and limited health facilities and the delay in the availability of histopathology results following biopsies [4].

This study highlights the challenges faced by the otorhinolaryngologist in our environment in the management of patients with head and neck malignant tumors and we recommend ways to improving the overall outcome of this disease.

## Methods

Approval was obtained for this retrospective study from the Ethical Clearance Committee of the Jos University Teaching Hospital.

The medical records of patients with histopathologically confirmed head and neck malignancies presenting to the otorhinolaryngology unit of the Jos University Teaching Hospital, Jos, Nigeria over a period of 10 years (July 1998 to June 2008) were analyzed for age, gender, social class of patient, time of presentation, nodal stage of disease at presentation, diagnostic protocol and the outcome of treatment. Patients were followed up throughout the study period and beyond for treatment outcome.

## Results

Eighty nine patients with head and neck malignancies aged between 23 years and 78 years were managed. There were 65 (73.0%) males and 24 (27.0%) females giving a male to female ratio of 2.7:1. Thirty four (38.2%) patients were in the 4^th^ decade of life (Table 1). Table 2 shows the representation of the various types of head and neck malignancies seen during the study period.

**Table 1:** Age distribution of patients with histopathologically confirmed head and neck malignancies presenting to the otorhinolaryngology unit of the Jos University Teaching Hospital, Jos, Nigeria over a period of 10 years (July 1998 to June 2008)

Age (years)	Frequency	Percentage
21–30	8	8.9
31–40	34	38.2
41–50	28	31.5
51–60	12	13.5
61–70	4	4.5
71–80	3	3.4
**Total**	**89**	**100**

**Table 2:** Frequencies of Head and neck cancers seen in a 99 patients with histopathologically confirmed head and neck malignancies presenting to the otorhinolaryngology unit of the Jos University Teaching Hospital, Jos, Nigeria over a period of 10 years (July 1998 to June 2008)

Cancer type	Frequency	Percentage
Nasopharyngeal	46	**51.7**
Oropharyngeal	6	6.7
Hypopharyngeal	2	2.2
Sino–nasal	24	27
Laryngeal	7	7.9
Parotid	4	4.5
**Total**	**89**	**100**

Thirty one (34.8%) patients presented with N1 nodal disease, 22 (24.7%) patients with N2a nodal disease and 36 (40.5%) patients with N2b nodal disease. The least clinical stage at presentation was a stage 2 disease and the most advanced stage at presentation was stage 4b.

The duration between disease onset and presentation for all the patients ranged from 3 to 18 months with 18 (20.2%) patients presenting within 6 months of onset of illness and 71 (79.8%) patients after 6 months. Thirty two (36%) patients visited the herbalist in the course of their illness before presenting to the hospital.

Thirty eight (42.7%) patients had cervical lymph node enlargement at presentation. Twenty six (68.4%) of these patients were subjected to open cervical lymph node biopsy by the general surgeons who were the first contact health personnel in our tertiary health center. The results from the pathologists which took a minimum of two weeks were reported as inconclusive and an advice for further evaluation of the patients to search for primaries in the head and neck. Twelve (31.6%) patients had FNAB with 9 positive for malignancy. All patients sustained scar following open biopsy, 15 (57.7%) had tumor spread.

Four (4.5%) patients were able to afford CT scan and these were of the high income group, 31(34.8%) patients belonged to the middle income group and 54 (60.7%) to the low income group. Eighty eight (99%) patients had endoscopic histologic biopsy of their primary tumors under general anesthesia and these were referred to a hospital about 200 kilometers away for chemo-radiotherapy because our hospital lacks facilities for radiotherapy. Four patients had total laryngectomy on account of laryngeal cancer before being referred for chemo-radiotherapy. One patient with nasopharyngeal cancer died before he could have endoscopic biopsy.

A total of 28 (32.6%) deaths were recorded- twenty two (79%) deaths were due to cancer, 5 (17.5%) from co-morbid medical conditions and in 1(3.5%), the cause of death was unknown. Ten patients were lost to follow-up.

## Discussion

Malignant tumors of the head and neck constitute one of the 10 most frequent malignancies worldwide with more than 500,000 new cases diagnosed annually [5]. They display considerable diverse variation in geographical distribution with varying epidemiological reports from different parts of the world for the different sub-sites in the head and neck [6,7]. The variation in incidence by sub-site is mostly related to the relative distribution of major risk/etiological factors such as tobacco or bidi smoking, tobacco or betel quid chewing, alcohol consumption, viruses, diet and familial risks [7]. The incidences of these diseases are higher in regions of the world where tobacco use and alcohol consumption is high [8]. The record of the incidence of head and neck malignancies in Nigeria is scanty [9].

Varying factors affect the outcome of this disease worldwide. In our environment, the interplay of several factors contributes to the eventual poor outcome in the management of patients with head and neck malignancies. These factors include late patient presentation, inaccessible health facilities, limited diagnostic and therapeutic tools, the delay in the availability of histopathology results following biopsies and the practice of subjecting patients with head and neck lymphadenopathy to open lymph node biopsy in the face of the afore mentioned [4].

Two major factors contribute to patients presenting late to hospitals in our environment and these are poverty and ignorance [10]. Majority of patients in our environment, especially those in the rural areas lack the financial means to access modern health facilities due to high poverty level and this is further compounded by harmful traditional beliefs and practices which makes them visit the herbalist for solutions to their health problems so that by the time they present to us, their tumors would have reached advanced stages and hence a poor outcome in management. This is buttressed by the fact that most of our patients belong to the low income group and therefore could not afford diagnostic facilities like CT scan even on presentation. The high income patients who presented to us had CT scan and they benefitted from this radiological diagnosis and are still alive following treatment.

Thirty eight (42.7%) of the patients in our series who had cervical lymphadenopathy at presentation were referred to the general surgeons in our center and were subjected to open cervical biopsies with attendant complications such as scar formation and tumor spread which was worsened by the delay in the availability of histological reports. Fine needle aspiration biopsy is preferable to open biopsy of a cervical lymph node for the reasons that there is no tumor spread, no inconvenient scar to distort future surgical intervention, no delay between diagnosis and treatment and its simplicity. When a diagnosis of malignancy cannot be made by needle biopsy, then an open biopsy can be done provided it can be followed by a frozen section and a concomitant definitive neck dissection if peroperative positive histological diagnosis is obtained [11]. Open cervical lymph node biopsy can alter patterns of lymphatic drainage for up to 1 year following surgery [12] and creates a scar which distorts future surgical intervention therefore altering the outcome of treatment [11].

Facilities for frozen section are not available in our center and delay in getting histopathology results from the open cervical biopsies and also following panendoscopy as seen in our study further compounds our patients’ problems as their tumors and disease process progresses further with eventual poor outcome.

There are only 5 functioning radiotherapy centers in Nigeria catering for a population of over 140 million. Our hospital lacks such facilities, which means that even when histological diagnosis is established, our patients still have to travel several kilometers to obtain treatment in another hospital with facilities for treatment. This also costs money and patients who cannot afford it end up not receiving treatment. We are of the opinion that majority of the patients we lost to follow-up fall into this category.

The challenges encountered in managing patients with head and neck malignant tumors in our environment are enormous but surmountable. The overall outcome of managing patients with head and neck malignant tumors can be improved upon and we therefore recommend the following: 1) Health workers should intensify efforts at educating individuals especially in the rural communities on these diseases specifying the importance of early hospital presentation and discouraging harmful traditional practices; 2) All patients with head and neck lymphadenopathy who present to any physician for diagnostic examination should undergo formal ENT staging and FNAB to avoid the problems of tumor spread and the reduction in consequent prognosis; 3) Even as prioritizing health care needs is a difficult task for developing nations in the face of limited and diminishing resources, our governments should wake up to their responsibilities. They have a pivotal role to play in capacity building and the development of our health care systems for adequate diagnosis and treatment that would ensure a better prognosis for patients with malignant head and neck tumors in our environment.

## Conclusion

The challenges encountered in managing patients with head and neck malignant tumors in our environment are surmountable. Health care providers and government should play committed roles in ensuring a better outcome in the management of these patients.
